# Protein Tyrosine Kinases: Their Roles and Their Targeting in Leukemia

**DOI:** 10.3390/cancers13020184

**Published:** 2021-01-07

**Authors:** Kalpana K. Bhanumathy, Amrutha Balagopal, Frederick S. Vizeacoumar, Franco J. Vizeacoumar, Andrew Freywald, Vincenzo Giambra

**Affiliations:** 1 Division of Oncology, College of Medicine, University of Saskatchewan, Saskatoon, SK S7N 5E5, Canada; amb850@mail.usask.ca (A.B.); franco.vizeacoumar@usask.ca (F.J.V.); 2 Department of Pathology and Laboratory Medicine, College of Medicine, University of Saskatchewan, Saskatoon, SK S7N 5E5, Canada; frederick.vizeacoumar@usask.ca (F.S.V.); andrew.freywald@usask.ca (A.F.); 3 Cancer Research Department, Saskatchewan Cancer Agency, 107 Wiggins Road, Saskatoon, SK S7N 5E5, Canada; 4 Institute for Stem Cell Biology, Regenerative Medicine and Innovative Therapies (ISBReMIT), Fondazione IRCCS Casa Sollievo della Sofferenza, 71013 San Giovanni Rotondo, FG, Italy

**Keywords:** protein kinase, receptor tyrosine kinase, non-receptor tyrosine kinase, leukaemia, small molecule inhibitor, therapeutic antibodies

## Abstract

**Simple Summary:**

Protein phosphorylation is a key regulatory mechanism that controls a wide variety of cellular responses. This process is catalysed by the members of the protein kinase superfamily that are classified into two main families based on their ability to phosphorylate either tyrosine or serine and threonine residues in their substrates. Massive research efforts have been invested in dissecting the functions of tyrosine kinases, revealing their importance in the initiation and progression of human malignancies. Based on these investigations, numerous tyrosine kinase inhibitors have been included in clinical protocols and proved to be effective in targeted therapies for various haematological malignancies. In this review, we provide insights into the role of tyrosine kinases in leukaemia and discuss their targeting for therapeutic purposes with the currently available inhibitory compounds.

**Abstract:**

Protein kinases constitute a large group of enzymes catalysing protein phosphorylation and controlling multiple signalling events. The human protein kinase superfamily consists of 518 members and represents a complicated system with intricate internal and external interactions. Protein kinases are classified into two main families based on the ability to phosphorylate either tyrosine or serine and threonine residues. Among the 90 tyrosine kinase genes, 58 are receptor types classified into 20 groups and 32 are of the nonreceptor types distributed into 10 groups. Tyrosine kinases execute their biological functions by controlling a variety of cellular responses, such as cell division, metabolism, migration, cell–cell and cell matrix adhesion, cell survival and apoptosis. Over the last 30 years, a major focus of research has been directed towards cancer-associated tyrosine kinases owing to their critical contributions to the development and aggressiveness of human malignancies through the pathological effects on cell behaviour. Leukaemia represents a heterogeneous group of haematological malignancies, characterised by an uncontrolled proliferation of undifferentiated hematopoietic cells or leukaemia blasts, mostly derived from bone marrow. They are usually classified as chronic or acute, depending on the rates of their progression, as well as myeloid or lymphoblastic, according to the type of blood cells involved. Overall, these malignancies are relatively common amongst both children and adults. In malignant haematopoiesis, multiple tyrosine kinases of both receptor and nonreceptor types, including AXL receptor tyrosine kinase (AXL), Discoidin domain receptor 1 (DDR1), Vascular endothelial growth factor receptor (VEGFR), Fibroblast growth factor receptor (FGFR), Mesenchymal–epithelial transition factor (MET), proto-oncogene c-Src (SRC), Spleen tyrosine kinase (SYK) and pro-oncogenic Abelson tyrosine-protein kinase 1 (ABL1) mutants, are implicated in the pathogenesis and drug resistance of practically all types of leukaemia. The role of ABL1 kinase mutants and their therapeutic inhibitors have been extensively analysed in scientific literature, and therefore, in this review, we provide insights into the impact and mechanism of action of other tyrosine kinases involved in the development and progression of human leukaemia and discuss the currently available and emerging treatment options based on targeting these molecules.

## 1. Introduction

**Figure 1:**
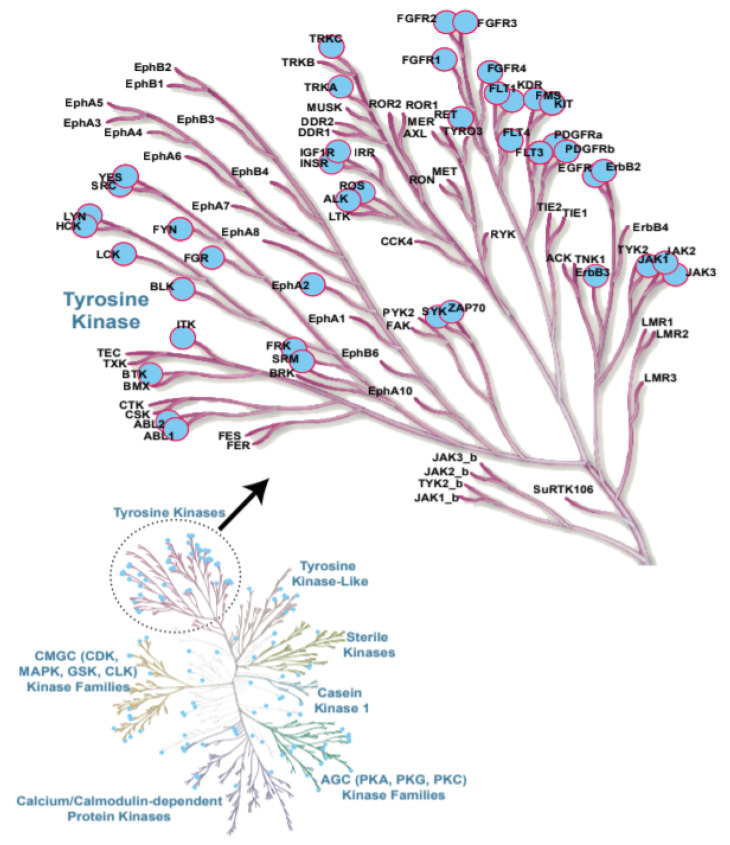
Protein kinases contributing to leukaemia are highlighted in blue. Examples of tyrosine kinases (TKs) involved in leukaemia are shown in the insert [21].

Protein kinases are a large family of enzymes that catalyse the phosphorylation of protein molecules. While kinases act as enzymes that transfer a phosphate group to a target molecule, phosphatases are enzymes with an opposite function to remove a phosphate group from a protein. These two enzymatic processes play a key role in the cell by regulating numerous signalling pathways in response to an external stimulus [1]. The human protein kinase gene family, which consists of 518 members, along with 106 pseudogenes, is the second largest enzyme family and the fifth-largest gene family in humans [2]. The deregulation of kinase activity results in the dramatic change of different cellular responses, such as cell cycle regulation, growth, differentiation, proliferation, survival, apoptosis, migration and several others [3]. As protein phosphorylation is one of the most common and important post-translational modifications, protein kinases are involved in multiple key regulatory pathways in the cell; deregulation or loss of their activities are often associated with the development of various types of cancers [4,5]. Further, genome-wide studies have provided key insights into the role of genetically inherited variants of specific kinases in several stages of cancer, including initiation, promotion, progression and recurrence [5,6]. Additionally, chromosomal mapping has provided evidence indicating that, out of the 518 protein kinase genes, 244 map to either disease loci or cancer amplicons, further highlighting the therapeutic potential of protein kinase inhibitors and their targets [2]. Several studies have linked protein kinases and deactivated phosphatases to multiple human malignancies owing to their genetic mutations and, also, chromosomal reshuffling patterns [7,8,9,10,11]. Since the abnormal regulation of protein kinases is strongly associated with human malignancies, including chronic and acute leukaemia, considerable efforts have been expended to dissect the functions of protein kinase-controlled signal transduction pathways in cancer [2,12]. Within leukemic malignancies, acute leukaemia are characterised by a rapid progression and are subdivided into two types based on their origins: Acute myeloid leukaemia (AML) and Acute lymphoblastic leukaemia (ALL), with about 80–85% of cases of the B-ALL and 15–20% of cases of the T-ALL [13,14] nature. While AML accounts for about 90% of all acute leukaemia in adults, ALL is the most common subtype found in childhood [15,16]. On the other hand, in chronic leukaemia, Chronic lymphocytic leukaemia (CLL) represents a heterogeneous disease with a variety of clinical outcomes that is characterised by the proliferation and accumulation of mature CD5^+^B cells in the blood, bone marrow and lymphoid tissues [17]. Chronic myelogenous leukaemia (CML) originates in a hematopoietic stem cell and is a chronic myeloproliferative disease that is characterised by an abnormal fusion gene (BCR-ABL1) on the Philadelphia chromosome in neoplastic cells [18]. Amongst the protein kinases, tyrosine kinases (TKs) [Discoidin domain receptor (DDRs), Erythropoietin-producing human haepatocellular (Eph) receptors, proto-oncogene c-Src (SRC), Spleen tyrosine kinase (SYK), Fms-like tyrosine kinase 3 (FLT3), Janus kinase (JAK), etc.] play a major role in haematopoiesis, and the deregulation of TK signalling has long been associated with the development of haematological malignancies [11,19,20]. The examples of TKs involved in leukaemia are highlighted in Figure 1 [21]. While TK mutations in AML account for FLT3 (30%), KIT (5%) and Janus kinase 2 (JAK2) (2%), the deregulated activity of ABL1 (within the BCR-ABL1 fusion, resulting from the t(9;22) (q34;q11) chromosomal translocation) drives the initiation and maintenance of CML [18,22,23]. Zeta chain of T-cell receptor-associated protein kinase 70 (ZAP-70), B lymphocyte kinase (BLK), JAK2, tyrosine-protein kinase (LYN) and neurotrophic Receptor Tyrosine Kinase 3 (NTRK3) are found to be the most commonly mutated kinases in CLL [24]. The abelson tyrosine-protein kinase 1 (ABL1) mutants and their therapeutic inhibitors have been extensively reported to play a critical role in leukaemia and discussed in detail in recent reviews [25,26,27].

This review is mainly focused on the impact and targeting of TKs not related to ABL1 but having a relevant role in the development and progression of different types of leukaemia. Emerging treatment options based on targeting these molecules and the currently available modulators of their activities are also being discussed.

## 2. Classification of TKs

The human genome contains 90 unique TK genes, out of which, 58 are receptor types, distributed into 20 subfamilies, and 32 are of nonreceptor types grouped into 10 subfamilies [2,28] (Table 1). Over the last 30 years, a major focus of research has been linked to multiple TKs owing to their importance in the development of different malignancies, including leukaemia, as well as nonmalignant disorders. TKs are important mediators of signal transduction, controlling cell proliferation, differentiation, migration, metabolism and programmed cell death, and are actively involved in all stages of neoplastic development and progression. The finding of the protein-tyrosine kinase (PTK) activities to be linked with viral transforming proteins and the *SRC* oncogene that has transforming nonreceptor tyrosine kinase (NRTK) activity led the way to the understanding of the role and significance of TKs in cancer [29,30]. Receptor tyrosine kinases (RTKs), a subclass of TKs, are key regulators of cellular processes and are essential regulators of signal transduction pathways, controlling a wide range of complex biological functions [31]. Due to their role as growth factor receptors and their ability to initiate complex network of signalling pathways in a wide variety of cell types, their abnormal activities, including gain-of-function mutations or receptor/ligand overexpression, have been associated with multiple cancers and, in particular, leukaemia [31,32]. In general, the activation of RTKs occurs through the formation of intermolecular dimerization in the presence of ligands, which, in turn, leads to the activation of their kinase function and phosphorylation of tyrosine residues in their cytoplasmic portions. In oncogenic conditions, gain-of-function mutations in RTKs often lead to their constitutive activation, even in the absence of a matching ligand.

**Figure 2:**
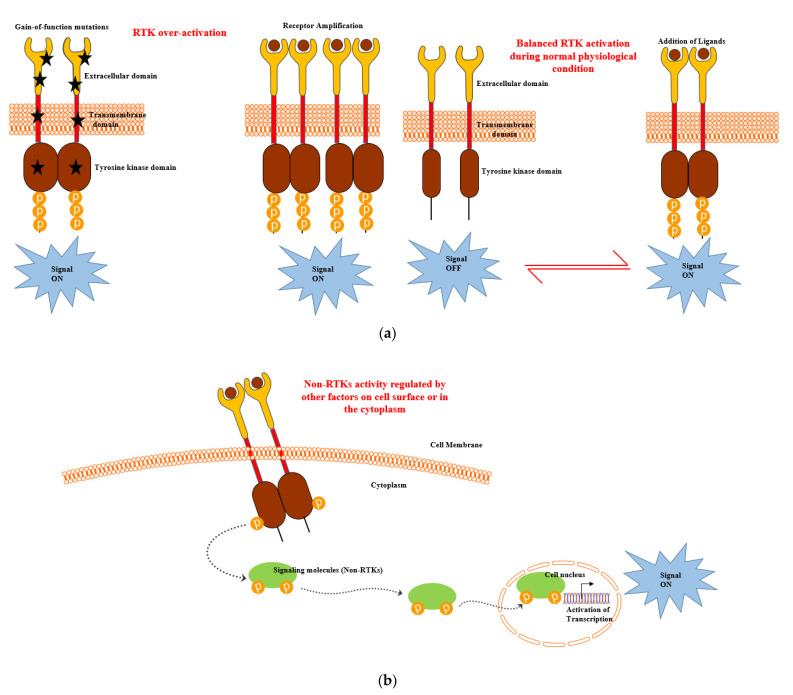
(**a**) Receptor tyrosine kinase (RTK) and (**b**) nonreceptor tyrosine kinase (NRTK) activation during leukaemia development and progression [10,31].

The overexpression of RTKs also frequently occurs in cancer as a consequence of genomic amplifications of RTK genes, which results in the increased accumulation of receptor molecules on the cell membrane and, ultimately, abnormally high level of signalling [31] (Figure 2a). Thus, deregulated RTKs have been reported to be responsible for the enhanced survival and apoptotic resistance in CLL [33,34,35,36,37]. NRTKs are cytosolic TK enzymes that lack a transmembrane domain. NRTKs exhibit significant structural variability due to the presence of a kinase domain, some protein–protein interacting domains (SH2, SH3 and PH domains) and additional signalling [10]. Therefore, the activation of NRTKs are regulated by other factors on the cell surface or in the cytoplasm integrating heterologous protein–protein interactions, thus enabling transphosphorylation (Figure 2b). NRTKs play crucial roles in the regulation of the immune system, cell growth, proliferation, differentiation, adhesion, migration and apoptosis. Mutations in NRTKs result in the formation of oncogenes [including ABL1, proto-oncogene tyrosine protein kinase c-Fes (FES), SRC, etc.] and leads to aberrant signalling. This has been reported in many forms of haematological malignancies, leading to their prolonged viability and overall survival [11].

In the following sections, we discuss the roles of individual TKs involved in the pathogenesis of leukaemia, including their roles in the onset and progression of different types of leukemic diseases and their targeting for therapeutic purposes.

### 2.1. FLT3

FLT3 belongs to the class III receptor TK family, which also includes platelet-derived growth factor receptor (PDGFR), macrophage colony-stimulating factor receptor (FMS) and stem cell factor receptor (KIT). FLT3 is important for the normal development of hematopoietic stem cells and the immune system [38,39]. In acute leukaemia, the expression of FLT3 is observed in 92% of AMLs and 27% of T-ALLs, and the overexpression of the FLT3 protein is usually found in 70–100% leukemic blast cells of AML patients. Mutations in the *FLT3* gene have been reported in many cases of AML [40,41]. The activating mutations in FLT3 can be found either in the internal tandem duplications (ITDs) of the juxta membrane domain or in the activation loop mutations (ALM) of the kinase domain (TKD) [42]. While the former interferes with the normal negative regulatory role of *FLT3* gene, the latter locks the receptor into an active conformation, resulting in a constitutively open ATP-binding pocket. In fact, FLT3 ITDs have been reported in 25–30% of patients with AML [43]. In a study conducted by Yamamoto et al. [44], the authors examined the D835 and ITD mutations of the *FLT3* gene in a total of 589 patients with haematological malignancies. The results of the study revealed several kinds of D835 missense mutations in 30 of the 429 (7.0%) AML and one of the 36 (2.8%) ALL patients. In addition, FLT3/ITD and FLT3/ALM mutations have also been reported in paediatric AML [45,46,47]. While the frequency of the former is approximately 15% lower than in adults [48,49,50], the latter is detected in 7% of cases, similar to that in adults [51]. Direct experiments have shown that the FLT3-ITD mutations alone are insufficient to cause AML but induce a myeloproliferative disease in a murine bone marrow transplant model. However, the combination of FLT3-ITD and promyelocytic leukemia protein (PML)/ retinoic acid receptor alpha (RARalpha) mutations induces an acute promyelocytic leukemia (APL)-like disease in recipient mice over a period of 7–23 weeks with 100% penetrance, favouring the hypothesis that AML is the consequence of cooperation between at least two classes of mutations [52,53]. The overexpression of the wild-type FLT3 collaborates with the *NUP98-HOX* gene fusions (*NUP98-HOXA10* and *NUP98-HOXD13*) to induce aggressive AMLs in mice [54,55]. Studies have also reported different gene rearrangements in CML and AML patients, examples of which include *NUP98/HOXA9* translocations in the combination ETV6/platelet-derived growth factor receptor beta (PDGFR β) or ABL1, respectively [56,57]. Of interest, a high FLT3 expression has also been associated with poor survival in KMT2A- AFF1+ ALL patients, further highlighting the relevance of FLT3 inhibitors for the treatment of ALL [58]. All these observations demonstrate the role of the FLT3 receptor in chronic and acute leukaemia and the identification of the mechanisms underlying its deregulation provides new opportunities in the treatment of these diseases [58,59].

### 2.2. KIT

KIT is a stem cell growth factor receptor involved in various functions of cell differentiation and survival, as well as melanogenesis, fertility and homeostasis [60]. It has been established that numerous malignancies such as mastocytosis, gastrointestinal tumours, melanomas and human mast cell leukaemia are the result of KIT mutations [61,62,63,64]. Almost 80% of AML cases have been found to have KIT proto-oncogene expression and support cell proliferation [65]. KIT has an additional role of improving the fibronectin attachment of the AML cells, leading to proliferative and antiapoptotic signals [66]. The structural organisation of KIT comprises a single transmembrane helix, a cytoplasmic juxtamembrane domain, a split kinase domain with a kinase insert and five immunoglobulin-like domains. It has been established that a major portion of mutations leading to disease development could be attributed to a few exons, including exon 17 encoding a kinase insert and exon 11 encoding a juxtamembrane domain and the extracellular exon 8 [67].

Amongst the different types of AML, core binding factor-AML has the maximum KIT mutations, with significant variations in reported cases. There has been evidence of KIT mutations having an adverse impact on relapse and survival in adult t(8;21) AML [68]. A study by Pollard et al. [69] reported a 20% prevalence of KIT mutations in their study population and suggested that, in paediatric patients with core binding factor-AML, KIT mutations did not have prognostic significance. This may be due to the fact that most studies of KIT mutations in children are smaller in size and reflect different settings of therapeutic treatments of AML patients. Molecular targeted therapies for leukaemia have led to the development of a vast array of drugs. KIT can be employed as a potential therapeutic target in two ways: first, by increasing the susceptibility of malignant cells to chemotherapeutic agents via synchronising the cells into the cell cycle and, secondly, by annulling the chemoresistance of leukaemia cells to apoptosis with the use of chemicals capable of blocking the KIT receptor activity [70,71]. Dasatinib is an FDA-approved small molecule compound that inhibits KIT [72]. Some other inhibitors with activity against KIT are SU6668 and SU5416, which are small-molecule indolinone-RTK inhibitors [73]. KIT inhibitors are further effective when used in combination with chemotherapy rather than alone, as cancers have multiple pathways of growth and proliferation [74]. With focused and in-depth research, targeting molecular abnormalities such as KIT can lead to less toxic treatments with higher efficiency than the conventional chemotherapy.

### 2.3. DDRs

DDRs belong to the RTK family and consist of two members DDR1 and DDR2, nonintegrin collagen-type receptors that signal in response to the binding of a collagen protein [75]. Although DDRs are mainly expressed during embryonic development, their role in adults is very limited. DDRs are either overexpressed and/or mutated in haematological cancers, their overexpression is correlated with a bad prognosis and their importance depends on the type and stage of each cancer type [76,77]. A study aimed at characterising gene expression signatures in ALL cells linked with known genotypic abnormalities in adult patients revealed that DDR1 was one among the kinases that was highly expressed in cases without molecular rearrangements, as well as in BCR/ABL1-positive ALL [78]. Barisione et al. [79] showed the correlation of DDR1 mRNA levels with CLL outcomes, suggesting the functional role of DDR1 in the development of this cancer. Moreover, B-ALL study cases in adults without *AFF4/AFF1* and *TCF3/PBX1* molecular rearrangements showed high transcriptional levels of the *DDR1* gene [78]. Tomasson et al. [80] reported DDR1 somatic mutations (DDR1^A803V^) in patients with de novo AML. These somatic mutation maps to the activation loop on its kinase domain, indicating that this DDR1 mutant may effectively disrupt kinase function. A high-throughput DNA sequencing study was conducted by Loriaux et al. to determine whether aberrantly activated TKs other than FLT3 and KIT receptors contribute to acute myeloid leukaemia (AML) pathogenesis [81]. Their results showed around 30 nonsynonymous sequence variations in 22 different kinases, and their analyses further identified five AML patients with nonsynonymous somatic mutations in the DDR1 juxtamembrane domain.

### 2.4. Eph Receptor Family

Eph receptors form the largest group of RTKs, and they are activated by ephrin ligands either attached to the cell membrane through glycosylphosphatidylinositol anchors or embedded there with their transmembrane domains. These receptors form an important cell communication system, with significant roles in both normal physiology and pathogenesis. There are fourteen Eph group members encoded in the human genome, which are further divided into A and B subtypes [82]. The EphA subtype consists of nine members (EPHA1–EPHA8 AND EPHA10) and are activated mostly by five ephrin-A ligands (EPHRIN-A1–EPHRIN-A5). The five EphB receptors (EPHB1–EPHB4 and EPHB6) interact predominantly with three ephrin-B ligands (EPHRIN-B1–EPHRIN-B3). The unusual feature that distinguishes them from most other RTKs is that Eph receptors bind to ephrin ligands expressed on the plasma membrane of a neighbouring cell and often form multimeric structures. Both the Eph receptors and ephrins can initiate signal transduction in each receptor- and ligand-presenting cell upon cell–cell contact formation, triggering both forward (through ephrin-ligated Eph receptors) and reverse (through Eph-bound ephrins) responses. The abnormal expression of the proteins of the Eph/ephrin system has been detected in many types of haematopoietic malignancies, and they have been reported to be either up- or downregulated based on the cancer type (Table 2). For example, EPHA3, originally identified in the LK63 pre-B ALL cell line, was later reported to be expressed in different cell lines of T-cell leukaemia and has been shown to induce both adhesive and repulsive responses in different cell types [83,84,85,86]. Altogether, the Eph-associated protein tyrosine phosphatase activity proved to not only control Eph receptor phosphorylation levels but, also, to switch the response of ephrin contact from repulsion to adhesion, thus playing a significant role in the pathology of some types of leukemic cells [86]. Several studies have shown the copy number variation of *EPHA*3 to be linked with various types of haematological malignancies, including AML, suggesting that these copy number variations could also be used as a diagnostic marker [87,88]. While aberrant EPHB4/EPHRIN-B2 expression has been found in different leukaemia and lymphoma cell lines [89], the overexpression of EPHB6 has been reported in both myeloid and lymphoid leukemic cells [90,91,92]. In a study by Nakanishi et al., the authors performed a screening study in K562 cells to determine whether ALL1 fusion proteins are involved in the regulation of *EPH* genes. These cells producing recombinant ALL1/AF4 or ALL1/AF9 fusion protein showed the transcriptional upregulation of *EPHA7* accompanied by ERK phosphorylation [93,94]. Thus, anti-EPHA7 antibodies may play a prominent role in leukaemia linked with these translocations.

### 2.5. SRC Kinases

The SRC family comprise genes encoding nine structurally related NRTKs, including SRC, BLK, FGR, FYN, HCY, LCK, LYN, YES and YRK, which play a major role in several physiological processes, like proliferation, angiogenesis, migration, differentiation, invasion and immune function [102]. At least some of them are distinctive proto-oncogenes, triggering unregulated cell proliferation as a result of mutation or overexpression in cancer. SRC activation may be due to interactions with cell membrane receptors [103] or a result of post-translational modifications and mutations [104], and we earlier reported that targeting the synthetic lethal interaction between the SRC kinase and the EPHB6 receptor may benefit cancer treatment, as this molecule is downregulated in multiple malignancies [105]. The role of the SRC kinase in CML was first established by Danhauser-Riedl et al. [106], who showed that LYN and haematopoietic cell kinase (HCK)-SRC-related kinases were activated, along with BCR-ABL1 fusion, in myeloid cells. Dos Santos et al. [107] found an interesting link between the LYN kinase and the mTOR pathway, asserting LYN as a therapeutic target for AML. It has been found that the SRC kinase uses different signalling pathways to induce both CML and B-ALL [108]. These results suggested that Philadelphia-positive acute leukaemia patients can be aided by the simultaneous co-inhibition of SRC and ABL1 kinases. Recent studies have given further insights into the importance of SRC kinases in different malignancies [109,110]. Weir et al. [109] found TL02-59 as one of the promising compounds in AML treatment, thereby establishing FGR as a novel therapeutic target in this malignancy. HCK was found to be involved in erythroid cell differentiation by being a part of the RAS-ERK and PI3K/AKT pathways. The elevated expression of HCK was observed in CD34+ haematopoietic stem and progenitor cell (HSPC) subsets of AML patients, paving the way for investigating HCK inhibitors [111]. Ingley [112] have reported the distribution of LYN throughout the plasma membrane and cytosol of AML cells with the most prominent expression amongst the SRC family members. Thus, the expression levels of HCK, FGR and LYN have a direct relation with the poor prognosis of the AML disease [110]. On the other hand, in B-CLL, LYN has been suggested as the only SRC kinase (compared to others, such as SRC, FYN, c-FGR and LCK) to be responsible for the abnormal cellular tyrosine phosphorylation and is the proximal kinase to initiate a signalling cascade in the BCR pathway [113]. Therefore, strategies to target BCR signalling as an emerging therapeutic approach have been well-discussed in the literature [114,115]. In brief, the BCR stimulation by antigen leads to the phosphorylation of the immunoreceptor tyrosine-based activation motifs (ITAMs) by LYN, following which, SYK is recruited and activated through tyrosine phosphorylation. The imbalanced activity within the BCR signalling axis has been responsible for the deregulation of the gene expression in CLL [116]. The LYN overexpression in CLL was linked with a significant downregulation of microRNA-337-5p. This suggests that the aberrant expression of microRNA-337-5p could be involved in the post-transcriptional regulation of LYN mRNA [117]. Taken together, all these studies indicate the potential importance of SRC kinase family members as novel targets for leukaemia treatment.

### 2.6. SYK Family

SYK is a cytoplasmic TK acting to couple BCR to intracellular signalling pathways in B lymphocytes [118]. SYK is a pro-survival factor, and therefore, its knockdown or inhibition leads to apoptosis in the cell subsets of CLL and ALL of the B cell lineage that express active SYK [119,120,121,122]. The observed effect might be due to the significant downstream reduction of major inflammatory mediators such as tumor necrosis factor alpha (TNFα), interleukins (ILs)-1, 6 and 18 that suggest the role of SYK in mediating the proinflammatory response [116]. Further, the inhibition of SYK mobilises CLL cells from tissue compartments to the blood by antagonising signals that restrain CLL cells within tissues, thus inducing cell death [123]. Understanding the molecular characterisations of these mobilised CLL cells and their sensitivity to chemotherapy play an important role in the identification and use of SYK inhibitors. SYK is expressed in a majority of AML patients and is a critical regulator of FLT3 in AML [124]. Efforts to use SYK inhibitors therapeutically in AML patients have shown promising results, especially in combination with standard chemotherapeutics such as cytosine arabinoside (AraC). Moreover, the inhibition of SYK has been reported to increase the sensitivity of leukaemia stem cells to AraC [125]. SYK is also reported to exhibit its cell survival function by signalling through BCR in haematological malignancies, implicating SYK as a promising target for the development of clinically relevant inhibitors [126,127,128].

### 2.7. JAK/STAT Signalling

**Figure 3:**
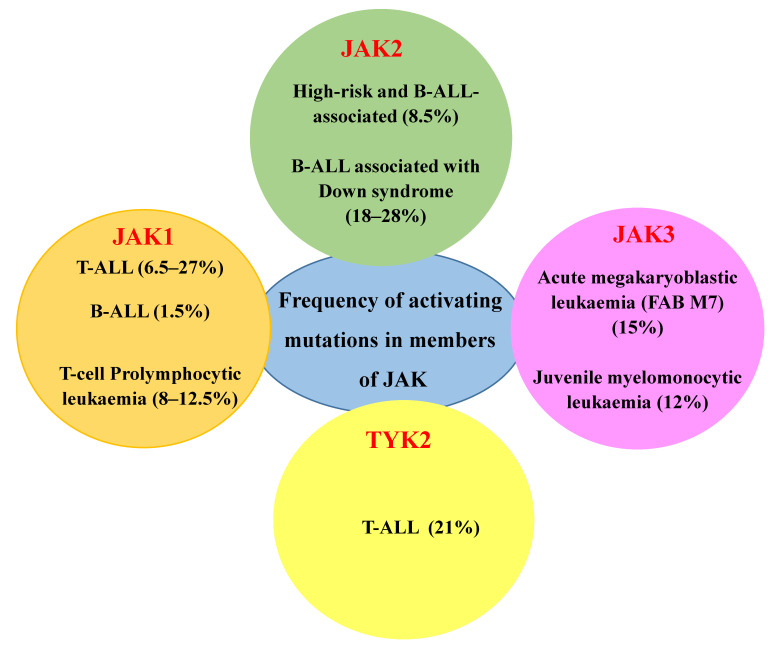
Frequency of activating mutations in members of JAK among different haematological malignancies [131].

JAKs are cytoplasmic TKs, including four family members: JAK1, JAK2, JAK3 and TYK2, which govern cell survival, proliferation, differentiation, haematopoiesis, immune response and apoptotic death response [129,130]. While the members of JAK1, JAK2 and TYK2 are ubiquitously expressed, JAK3 expression is exclusively restricted to the haematopoietic lineage. The frequency of alterations in JAK members among different haematological malignancies is depicted in Figure 3.

Mutations in the JAK/STAT signalling pathway are hallmark features of several types of leukaemia, such as T-cell prolymphocytic leukaemia, paediatric-ALL and adult T-cell lymphoblastic leukaemia, and the identification of these mutations paves the way for understanding the pathogenesis and in developing targeted therapies for these aggressive malignancies [132,133,134,135]. Chimeric proteins with constitutive kinase activity produced by fusion genes resulting from chromosomal translocations, such as *ETV6-JAK2*, have also been reported in human leukaemia [136]. The *ETV6* gene located on the chromosome 12p13 has been reported to be involved in chromosomal translocations observed in a variety of human leukaemia since 1994 [56,137,138]. Inherent mutations are the most common factors attributed towards the development of malignancies, especially in myeloproliferative neoplasms. It has been reported that the acquisition of the constitutively active Janus kinase 2 (JAK2) V617F mutant occurs in 39–57% of primary myelofibrosis, 41–72% of essential thrombocytosis, 81–99% of Polycythaemia vera and <5% of acute myeloid leukaemia patients [139]. The activation of the transcription factor STAT5 is required for JAK2 V617F-mediated transformation, and therefore, the development of drugs that inhibits STAT5 can be an effective treatment option for myeloproliferative neoplasia [140]. A comprehensive sequence analysis by Vincente et al. identified mutations and copy number variations in IL7R-JAK signalling pathway members among 27.7% of T-ALL samples screened, with JAK3 mutations being the most frequent among others [141,142]. Another study by Degryse and Cools established the involvement of JAK kinases in T-ALL by affirming that the inhibition of both JAK-1 and JAK-3 can be used for T-ALL treatment [143]. This study also provided insights into the use of numerous JAK inhibitors that are already available to be potentially repurposed for T-ALL therapy [143]. The JAK/STAT signalling pathway is activated in response to the AML infiltration, and the inhibition of JAK1/2 resulted in significant anti-leukaemic activity in vitro [144]. In most leukaemia cases, JAK-2 has been the key regulator. However, JAK-3 activating mutations have also been observed in natural killer cell/T-cell lymphoma (35.4%) [145]. Findings have backed the persistent involvement of JAK-STAT pathways in different malignancies, which can open up novel targeted therapeutic approaches aiming to manipulate molecules associated with JAK kinases.

## 3. TK Inhibitors in Leukaemia and Lymphoma Treatment

Both leukaemia and lymphoma affect circulating leukocytes and bone marrow; however, lymphoma in general has higher survival rates than leukaemia. Thus, AML has only around 10% five-year survival rates in adults above 60 years of age and is accountable for more than half of leukaemia-related deaths in children. With novel chemical compounds emerging in cancer treatment every year, there has been a substantial contribution of small molecule inhibitors towards the development of new targeted therapies. Overall, small molecule inhibitors have been effective in suppressing disease progression in many types of cancers [146]. Protein kinases, as described previously, are key regulators of multiple aspects of cell biology, and their aberrant behaviours often lead to serious pathological conditions. Dysfunction in protein kinase signalling pathways has a major role in the development of different types of cancers, as well as cardiovascular diseases [3,147]. Kinase inhibitors are consequently amongst the most sought-after compounds in the development of targeted therapies with small molecule inhibitors. A brief account of different types of inhibitors based on the target site and mechanism of action is summarised in (Table 3).

The inhibitors are classified as covalent/irreversible and noncovalent/reversible, based on their binding to kinases. The covalent inhibitors largely bind to the lysine or cysteine residues around the ATP-binding site, and it has been proposed that such molecules are possibly toxic in nature [149]. Based on the ability to bind to the hinge region, noncovalent inhibitors are categorised into type 1, type 2 and type 3 kinase inhibitors. The unique binding sites of type 3 inhibitors enable them to have a higher degree of selectivity when compared to type 1 and type 2 inhibitors [150,151]. A major portion of the inhibitors are those that target the ATP-binding site, whereas the remaining ones target selected allosteric sites. Certain attributes of a kinase inhibitor, such as pharmacokinetic and pharmacodynamic properties and the degree of oncogenic addiction to the specific kinase, determine their therapeutic efficacy [152]. Small molecule inhibitors were first synthesised as early as 1984 by Hidaka et al., who synthesised naphthalene sulphonamide, which acted as the basis for the development of next-line molecules [153]. Kinase inhibitors such as imatinib, previously known as ST1571, an ABL1 TK inhibitor, has been proven to be quite effective as an anti-leukaemic agent [154]. Imatinib became the first economically successful FDA-approved small molecule protein kinase inhibitor for the treatment of CML patients in 2001 [155]. Consequently, there has been a steady increase in the number of FDA-approved kinase inhibitors, with more than 70 drugs making it to the list now. Table 4 depicts different drugs/small molecule inhibitors for protein TKs that have been approved by the FDA for clinical use.

Consistent with the important role of TK signalling in malignancy, amongst their inhibitors approved for clinical protocols, most are being used for treating various types of cancers [149]. For example, the advent of imatinib treatment has been revolutionary in patients with CML, assisting in their management and improving their overall survival. Other TK inhibitors, including dasatinib, nilotinib and bosutinib, are mostly prescribed in cases where the patient does not respond to imatinib. Research findings have strongly supported the fact that inhibitors of protein kinases have proven to be extremely useful in targeted therapy against different types of leukaemia, either alone or in combination with other drugs [167]. To give a few examples, midostaurin was approved in the United States of America for the treatment of adult patients with newly diagnosed, FLT3 mutation-positive AML, aggressive systemic mastocytosis, systemic mastocytosis with associated haematological neoplasm or mast cell leukaemia [164]. In addition, midostaurin was also used in combination with standard cytarabine and daunorubicin induction and cytarabine consolidation [164]. Cytarabine can also be used in combination with sunitinib against AML cell lines with the FL3-ITD mutation and tandutinib for the treatment of AML patients [168]. Trametinib, a MEK inhibitor, has been reported to exhibit strong synergy when combined with midostaurin in cells with mutated and wild-type *FLT3* [169]. Moreover, a recent study in the first-line setting for CLL has demonstrated the efficacy of single-agent ibrutinib, which significantly prolonged progression-free survival compared with chlorambucil, with an 85% reduction in the risk of disease progression or death [170]. The further investigation of functions of TKs in cancer cells can support the development of new inhibitory therapeutic compounds. Certain compounds, such as BTK inhibitor ibrutinib, have also been proven to be effective against mantle cell lymphoma patients. Mantle cell lymphoma has a destructive clinical progression with no effective therapy; however, ibrutinib shows complete response rates ranging from 19–23% in this malignancy. Further, second-generation BTK inhibitors have emerged, such as acalabrutinib, tirabrutinib and zanubrutinib, which are superior to ibrutinib in terms of efficacy and toxicity profiles [171]. Although the list of TK inhibitors has significantly increased over the past decades, the main challenge with these drugs is that they mostly have limited specificity and efficiency, which increases the risk of undesirable off-target effects and emerging evading pathways. This might ultimately lead to severe side effects in the patients and to the development of treatment-resistant leukaemic cell populations. Thus, mutations of protein kinases, instability of the inhibitors, residual and acquired drug resistance, general toxicity and off-target effects are some of the major concerns associated with the action of even the last-generation drugs [168]. Another important limitation of kinase inhibitors is the out-of-pocket expenses associated with their usage. For example, continuous therapy with ibrutinib used in the treatment of some lymphomas costs about USD 150,000 per year for each patient, and treatment with imatinib (standard therapy in CML) costs roughly USD 100,000 per patient per year lifelong. It has been observed that post the entry of generic imatinib, there has been a steady decline in its expenditure, but there is still a sustained surge in dasatinib and nilotinib expenditure in patients with CML [172]. The development of new-generation drugs with more specificity and less side effects greatly benefit patients. New-generation drugs are preferred over the older versions due to their ability to reduce serious limitations, such as low potency and specificity in addition to their better ability to cross the blood–brain barrier. An example would be the development of nilotinib, a second-generation drug with enhanced properties in its structure leading to higher binding affinity of the drug to ABL1, when compared to its first-generation counterpart imatinib. Imatinib was the first drug to specifically target the ABL1 kinase, and nilotinib works with lesser harmful side effects [173]. Thus, the development of next-generation kinase inhibitors based on the advances in cancer biology and structural biology research helps in generating more effective compounds with better therapeutic windows.

## 4. Conclusions

The action of TKs is indispensable in cell regulation, with functions ranging from controlling growth, cell cycle, adhesion and migration to governing apoptotic responses. With evidence of their contributions to human malignancies, these molecules are popular targets in drug discovery and in existing cancer treatment protocols. In a nutshell, we discussed here the role of TKs in the development and progression of different types of leukaemia. The overexpression and mutation of both receptor and NRTKs lead to impaired regulation in pathways, often supporting the initiation and progression of leukaemic diseases. FLT3 and KIT have been implicated in cases of acute and chronic leukaemia, opening up the prospects of treatment with higher efficacy if the underlying mechanisms of deregulation are fully investigated. While DDRs have been shown to be involved in lymphocytic leukaemia, mostly, SRC kinases are potential drivers in both lymphocytic and myeloid leukaemia. Eph receptors and their ligands are known to have important functions in physiology and pathogenesis; their upregulation or downregulation is associated with multiple haematologic malignancies. SYK is another kinase associated with intracellular signalling pathways in B cells contributing to leukaemia and lymphomas. JAKs are key regulators within the immune system and haematopoiesis. Therefore, their inherent mutations can often lead to different types of cancer, including ALL and Non-Hodgkin’s lymphoma. With the advent of targeted therapies and the supporting advanced technology, medicine has witnessed a surge in novel and more effective treatment options for cancer. Small molecule compounds, especially TK inhibitors, have been developed, with more agents being granted FDA approval every year. Further detailed analyses of the action of TKs in leukaemia and screening new compounds targeting these molecules should further facilitate detecting effective and less toxic inhibitors aimed at combating leukaemic diseases either alone or in combination with conventional chemotherapies for haematological malignancies.

## Figures and Tables

**Table 1:** Receptor and nonreceptor Classes of tyrosine kinase (TK) families.

RTKs	NRTKs
ALK receptor family (e.g., LTK and ALK) DDR family (e.g., DDR1, DDR2) EGF receptor family (e.g., EGFR, HER2, HER 3, HER4) Eph receptor family (e.g., EphA1-8 and EphA10, EphB1-B4 and EphB6) FGFR family (e.g., FGFR1- FGFR 4) INSR and IGF1 receptor (IGF1R) (e.g., IGF1, IGF2, IGF1R, IGF2R), IGFBP1- IGFBP 6)) MET receptor family (Met) MuSK receptor family NOK receptor family PDGFR family (e.g., alpha-type and beta-type PDGFRs) PTK7receptor family RET receptor family ROR receptor family ROS1 receptor family RYK family TAM receptor family (e.g., TYRO3, AXL, and MER) TIE receptor family (TIE1, TIE2) TRK receptor family (NTRK1, NTRK2, NTRK3) Uncharacterised (RTK106) VEGFR family (VEGFR-1/FLT-1 (FMS-like TK, VEGFR-2/KDR/FLK-1)	ABL1 and BCR-ABL family (e.g., ABL1, ARG) ACK (e.g., ACK1, TNK1) CSK (e.g., CSK, MATK) FAK family (e.g., FAK, PYK2) FES family (e.g., FES, FER) FRK (e.g., FRK, BRK, SRMS) JAK (e.g., JAK1- JAK 3, TYK2) SRC family (e.g., SRC, FGR, FYN, YES1, BLK, HCK, LCK, LYN) SYK family (e.g., SYK, ZAP70) TEC family (e.g., REC, BMX, BTK, ITK, TXK)

Note: Abbreviations within the table: RTKs- Receptor tyrosine kinases, NRTKs- nonreceptor tyrosine kinases, ALK—Anaplastic lymphoma kinase, FGFR—Fibroblast growth factor receptor, INSR—Insulin receptor, IGF—Insulin-like growth factor, IGFR—Insulin-like growth factor receptor, IGFBP—Insulin like growth factor binding protein, MET—Mesenchymal–epithelial transition factor, MuSK—Muscle-Specific Kinase, NOK—Novel oncogene with kinase domain, PTK7—Tyrosine protein kinase-like 7, RET: Rearranged during transfection, ROR—Retinoic acid-related orphan receptors, ROS1—Proto-oncogene tyrosine protein kinase, RYK—Related to receptor tyrosine kinase, TAM—Tumour-associated macrophage, TYRO3—TYRO3 Protein Tyrosine Kinase, AXL—AXL Receptor Tyrosine Kinase, MER—MER Proto-oncogene tyrosine kinase, TIE—Tyrosine kinase with immunoglobulin-like and EGF-like domains, TRK—Tropomyosin receptor kinase, VEGFR—Vascular endothelial growth factor receptor, FMS—proto-oncogene that codes for the macrophage colony-stimulating factor (CSF1) receptor, CSF—colony-stimulating factor, FLT—Fms-like receptor tyrosine kinase, KDR—Kinase insert domain receptor, FLK—Fetal liver kinase, ARG—Abelson-related gene, TNK—tyrosine kinase nonreceptor, CSK—C-terminal src kinase, MATK—Megakaryocyte-associated tyrosine kinase, FAK—Focal adhesion kinase, PYK2—Pyruvate kinase 2, FES—feline sarcoma oncogene, FER—fer (fps/fes-related) tyrosine kinase (phosphoprotein NCP94), FRK—fyn-related kinase, BRK—Breast tumour kinase, SRMS—Src-related kinase lacking C-terminal regulatory tyrosine and N-terminal myristylation sites, BLK—B lymphocyte kinase, FGR—Gardner-Rasheed feline sarcoma viral (v-fgr) oncogene homologue, HCK—Haematopoietic cell kinase, LCK—Lymphocyte-specific protein tyrosine kinase, YES1—YES Proto-Oncogene 1, Src Family Tyrosine Kinase, BMX—BMX nonreceptor tyrosine kinase, ITK—Interleukin-2-inducible T-cell kinase, TEC—Tyrosine-protein kinase Tec and TXK—Tyrosine-protein kinase TXK.

**Table 2:** Examples of the regulation of Eph family members in haematopoietic malignancies.

Eph Family	Cancer Type	Regulation	Reference
EPHB4	AML ALL	Up Down	Merchant et al. [95] Kuang et al. [96]
EPHB1	AML	Down	Kampen et al. [97]
EPHB6	T-ALL	Up	El Zawily et al. [98]
EPHA4, EPHB2 AND EPHB4	AML	Down	Wrobel et al. [99], Tyner et al. [100]
EPHA7	AFF4-associated leukemia Follicular Lymphoma	Up Down	Nakanishi et al. [93] Oricchio et al. [101]
EPHA3	ALL, AML, CLL, CML	Down	Guan et al. [88], Walter et al. [87]
EPHRIN-A4	CLL	Up	Alonso-C et al. [92]

**Table 3:** Classification of protein kinase inhibitors.

Type	Subtypes	Target Site	Diseases	Examples	Reference
Type I	A and B, with long and short residence times respectively	Binds to the ATP-binding pocket in the active conformation	ALL, CML	Bosutinib, Gefitinib,	Roskoski, 2016 [148]
Type I1/2	A and B, with long and short residence times respectively	Binds to the aspartate-phenylalanine-glycine (DFG) motif in inactive conformation	CML, ALL, Hairy cell leukaemia	Vemurafenib, Sunitinib	
Type II	A and B, with long and short residence times respectively	Occupies part of ATP-binding pocket and forms hydrogen bonds with the hinge region	CML	Sorafenib, Imatinib	
Type III	-	Occupies a site next to the ATP-binding pocket (Allosteric)	Relapsed/ Refractory AML	Cobimetinib, Trametinib	
Type IV	-	Undergoes a reversible interaction outside the ATP pocket and offers selectivity against targeted kinases (Substrate-directed/Allosteric)	CML	GNF-2	
Type V	-	Binds to two different regions of the protein kinase domain (Bivalent)	AML	4– Anilinoquinazoline	
Type VI	-	Binds covalently (irreversible) to their protein kinase target	CLL	Afatinib, Ibrutinib	

**Table 4:** List of a few examples of small molecule protein kinase inhibitors approved for clinical use.

Small Molecule Inhibitor	Target Kinase	Disease/Cancer	Approved in Year	Reference
Imatinib	ABL1, c-KIT, PDGFR	CML	2001	[156]
Nilotinib	ABL1	CML	2007	[157]
Bosutinib	ABL1, SRC	CML	2012	[158]
Ponatinib	SRC, ABL1	CML, ALL	2012	[159]
Radotinib	ABL1, PDGFR	CML	2012	[160]
Ibrutinib	BTK	Mantle cell lymphoma, CLL	2013	[161]
Idelalisib	PI3Kdelta	CLL	2014	[162]
Acalabrutinib	BTK	CLL, Mantle cell lymphoma	2017	[163]
Midostaurin	FLT3, KIT	AML, Mastocytosis	2017	[164]
Gilteritinib	FLT3, AXL	AML	2018	[165]
Zanubrutinib	BTK	Mantle cell lymphoma	2019	[166]

Note: The table is by no means comprehensive.

## Data Availability

No new data were created or analyzed in this study. Data sharing is not applicable to this article.

## Abbreviations

ABL1	Abelson tyrosine-protein kinase
ACK	Activated Cdc42-associated kinase
ALL	Acute lymphoblastic leukaemia
ALM	Activation loop mutations
AML	Acute myeloid leukaemia
APL	Acute promyelocytic leukaemia
ATP	Adenosine triphosphate
B- ALL	B-cell acute lymphoblastic leukaemia
BCR	Breakpoint cluster region
BTK	Bruton’s tyrosine kinase
CDC	Cell division cycle
CDK	Cyclin-dependent kinases
c-KIT	CD117, also called KIT or C-kit receptor
CLL	Chronic lymphocytic leukaemia
c-Met	mesenchymal–epithelial transition factor
CMGC	CDK/MAPK/GSK/CDK-like kinase group
CML	Chronic myelogenous leukaemia
DDR	Discoidin domain receptor
EGF	Epidermal growth factor
EGFR	Epidermal growth factor receptor
EPH	erythropoietin-producing human haepatocellular receptors
Eph A	Ephrin type A receptors
EphB	Ephrin type B receptors
ERBB2	Receptor tyrosine protein kinase erbB-2
ERK	extracellular signal-regulated kinase
FDA	Food and drug administration
FGFR	Fibroblast growth factor receptor
FGR	Gardner-Rasheed feline sarcoma viral (v-fgr) oncogene homologue
FYN	FYN oncogene related to SRC, FGR and YES
GSK	Glycogen synthase kinase
HCK	Haematopoietic cell kinase
HDAC	Histone deacetylase
HER	Human epidermal growth factor receptor
HSPC	Haematopoietic stem and progenitor cell
ITAMs	Immunoreceptor tyrosine-based activation motifs
ITDs	Internal tandem duplications
JAK	Janus kinase
JAK-STAT	Janus kinase signal transducer and activator of transcription
JMML	Juvenile myelomonocytic leukaemia
MAPK/ERK	Mitogen-activated protein kinases/Extracellular signal-regulated kinases
mTOR	The mammalian target of rapamycin
NRTK	Nonreceptor tyrosine kinase
NSCLC	Non-small cell lung carcinoma
PARP	Poly (ADP-ribose) polymerase
PDGFR β	Platelet-derived growth factor receptor beta
PI3K/AKT	Phosphatidylinositol 3-kinases/Protein kinase B
PML	Promyelocytic leukaemia
PTP	Protein tyrosine phosphatase
PTPN1	Tyrosine protein phosphatase nonreceptor type 1/protein-tyrosine phosphatase 1B (PTP1B)
PTPN2	Tyrosine protein phosphatase nonreceptor type 2
RAF	RAF proto-oncogene serine/threonine protein kinase
RARA	Retinoic acid receptor alpha
RTK	Receptor tyrosine kinase
SRC	Rous sarcoma oncogene cellular homologue/Proto-oncogene tyrosine protein kinase Src
STAT5	Signal transducer and activator of transcription 5
SYK	Spleen-associated tyrosine kinase
T-ALL	T-cell acute lymphoblastic leukemia
ETV6	Translocation-ETS-leukemia/ETV6
TKD	Tyrosine kinase domain
TKI	Tyrosine kinase inhibitors
TKs	Tyrosine Kinases
TNFα	Tumor necrosis factor alpha
TYK2	Tyrosine kinase 2
ZAP-70	Zeta chain of T-cell receptor-associated protein kinase 70
